# Zika Virus Infects Trabecular Meshwork and Causes Trabeculitis and Glaucomatous Pathology in Mouse Eyes

**DOI:** 10.1128/mSphere.00173-19

**Published:** 2019-05-08

**Authors:** Pawan Kumar Singh, Ramesh B. Kasetti, Gulab S. Zode, Anju Goyal, Mark S. Juzych, Ashok Kumar

**Affiliations:** a Department of Ophthalmology, Visual and Anatomical Sciences/Kresge Eye Institute, Wayne State University School of Medicine, Detroit, Michigan, USA; b The North Texas Eye Research Institute and the Department of Pharmacology and Neurosciences, University of North Texas Health Science Center, Fort Worth, Texas, USA; University of Chicago

**Keywords:** RGC, Zika virus, axonal transport, eye, glaucoma, intraocular pressure, ocular infection, optic nerve, retina, trabeculitis

## Abstract

Zika virus (ZIKV) infection during pregnancy leads to devastating fetal outcomes, including neurological (microcephaly) and ocular pathologies such as retinal lesions, optic nerve abnormalities, chorioretinal atrophy, and congenital glaucoma. Only clinical case reports have linked ZIKV infection to causing glaucoma, a major blinding eye disease. In the present study, we have investigated the role of ZIKV in glaucoma pathophysiology using *in vitro* and *in vivo* experimental models. We showed that human primary trabecular meshwork (Pr. TM) cells, as well as a human GTM3 cell line, were permissive to ZIKV infection. ZIKV induced the transcription of various genes expressing pattern recognition receptors (*TLR2*, *TLR3*, and *RIG-I*), cytokines/chemokines (*TNF-α*, *IL-1β*, *CCL5*, and *CXCL10*), interferons (*IFN-α2*, *IFN-β1*, and *IFN-γ*), and interferon-stimulated genes (*ISG15* and *OAS2*) in Pr. TM cells. ZIKV infection in IFNAR1^−/−^ and wild-type (WT) mouse eyes resulted in increased intraocular pressure (IOP) and the development of chorioretinal atrophy. Anterior chamber (AC) inoculation of ZIKV caused infectivity in iridocorneal angle and TM, leading to the death of TM cells in the mouse eyes. Moreover, anterior segment tissue of infected eyes exhibited increased expression of inflammatory mediators and interferons. Furthermore, ZIKV infection in IFNAR1^−/−^ mice resulted in retinal ganglion cell (RGC) death and loss, coinciding with optic nerve infectivity and disruption of anterograde axonal transport. Because of similarity in glaucomatous pathologies in our study and other experimental glaucoma models, ZIKV infection can be used to study infectious triggers of glaucoma, currently an understudied area of investigation.

**IMPORTANCE** Ocular complications due to ZIKV infection remains a major public health concern because of their ability to cause visual impairment or blindness. Most of the previous studies have shown ZIKV-induced ocular pathology in the posterior segment (i.e., retina) of the eye. However, some recent clinical reports from affected countries highlighted the importance of ZIKV in affecting the anterior segment of the eye and causing congenital glaucoma. Because glaucoma is the second leading cause of blindness worldwide, it is imperative to study ZIKV infection in causing glaucoma to identify potential targets for therapeutic intervention. In this study, we discovered that ZIKV permissively infects human TM cells and evokes inflammatory responses causing trabeculitis. Using a mouse model, we demonstrated that ZIKV infection resulted in higher IOP, increased RGC loss, and optic nerve abnormalities, the classical hallmarks of glaucoma. Collectively, our study provides new insights into ocular ZIKV infection resulting in glaucomatous pathology.

## Abstract (precis)

Ocular complications due to ZIKV infection remains a major public health concern because of their ability to cause visual impairment or blindness. Most of the previous studies have shown ZIKV-induced ocular pathology in the posterior segment (i.e., retina) of the eye. However, some recent clinical reports from affected countries highlighted the importance of ZIKV in affecting the anterior segment of the eye and causing congenital glaucoma. Because glaucoma is the second leading cause of blindness worldwide, it is imperative to study ZIKV infection in causing glaucoma to identify potential targets for therapeutic intervention. In this study, we discovered that ZIKV permissively infects human TM cells and evokes inflammatory responses causing trabeculitis. Using a mouse model, we demonstrated that ZIKV infection resulted in higher IOP, increased RGC loss, and optic nerve abnormalities, the classical hallmarks of glaucoma. Collectively, our study provides new insights into ocular ZIKV infection resulting in glaucomatous pathology.

## INTRODUCTION

Zika virus (ZIKV) is a mosquito-borne flavivirus mostly transmitted by *Aedes* mosquitoes; however, cases of sexual transmission and transmission via blood transfusions have been also reported (1–5). ZIKV gained global attention due to its pregnancy-related complications leading to microcephaly and other devastating congenital malformations such as miscarriage, hydrocephaly, and fetal death, now collectively named congenital Zika syndrome (2, 6–8). Congenital ZIKV infection is associated with not only neurological but also with ocular, hearing, and musculoskeletal abnormalities (9–12). ZIKV has been reported to cause retinal lesions, focal pigmented mottling, microphthalmia, hemorrhagic retinopathy, retinal vasculitis, optic nerve (ON) abnormalities, and chorioretinal atrophy in the eyes of newborns born from mothers infected during pregnancy (5, 6, 11–17). ZIKV has also been known to cause acute ocular infection, including conjunctivitis, iridocyclitis, and chorioretinitis in adults (14). While most of the ocular manifestations have been restricted to the posterior segment only, recent clinical case reports from Brazil, Colombia, and Venezuela have raised concern for anterior segment involvement, primarily in causing glaucoma (18–21). At present, glaucoma is a rare consequence of congenital infection and has not been described among infants exposed to any infection during gestation.

Studies from our lab and others have shown that ZIKV infects a variety of retinal (6, 12, 17, 22–24), corneal (25), and other cell types, including neuronal cells (26, 27); placental cells (28, 29); skin fibroblasts, keratinocytes, and dendritic cells (30); and endometrial stromal cells (31). *In vivo* studies using local as well systemic infection in mouse models have demonstrated that ZIKV causes a variety of ocular pathologies and infects various parts of the eye including ciliary body, iris, conjunctiva, retina, and optic nerve head (6, 12, 17). Earlier studies have reported that adult C57BL/6 wild-type (WT) mice are resistant to neurotropic viruses including ZIKV, possibly due to the presence of protective blood-retinal barrier (BRB). BRB reduces predisposition of mature neurons to infection, thereby blocking the ZIKV capability to block the antiviral interferon response which can clear the virus (6, 32). Therefore, most studies have either utilized immunocompromised mouse models (IFNAR1^−/−^ and AG129) (11, 17, 33–36) or directly inoculated ZIKV into the desired tissue/organ (37–40). Recently, Manangeeswaran et al. (6) developed a mouse model using neonatal pups from WT mice (1 day postbirth) and demonstrated that ZIKV preferentially infects cornea and retina, resulting in chorioretinal atrophy. They also found that ZIKV infected retinal ganglion cells (RGCs) and cells in the inner nuclear layer, resulting in focal retinitis and optic nerve head infection, and caused thinning of the outer plexiform layer (6). In a nonhuman primate macaque model, ZIKV infection was shown to cause the fusion of iris with the posterior cornea and lack of maturation of iridocorneal angle with anterior segment dysgenesis in the fetus (41). Despite several studies using various ocular infection models and clinical cases from affected countries, there is a lack of studies revealing the role of ZIKV in glaucoma induction. Therefore, it is imperative to investigate the link between ZIKV and glaucoma pathogenesis for better understanding of the disease and to identify potential targets for therapeutic intervention.

In the present study, we investigated ZIKV infectivity in the trabecular meshwork (TM) using human primary TM cells as well as immortalized human TM cell line GTM3. We also investigated the role of ZIKV in glaucoma induction using wild-type (WT) and IFNAR1^−/−^ mouse models. Our results demonstrated for the first time that ZIKV permissively infects TM cells and induces trabeculitis and glaucoma-related pathology in the mouse model.

## RESULTS

### ZIKV infects TM cells.

**FIG 1:**
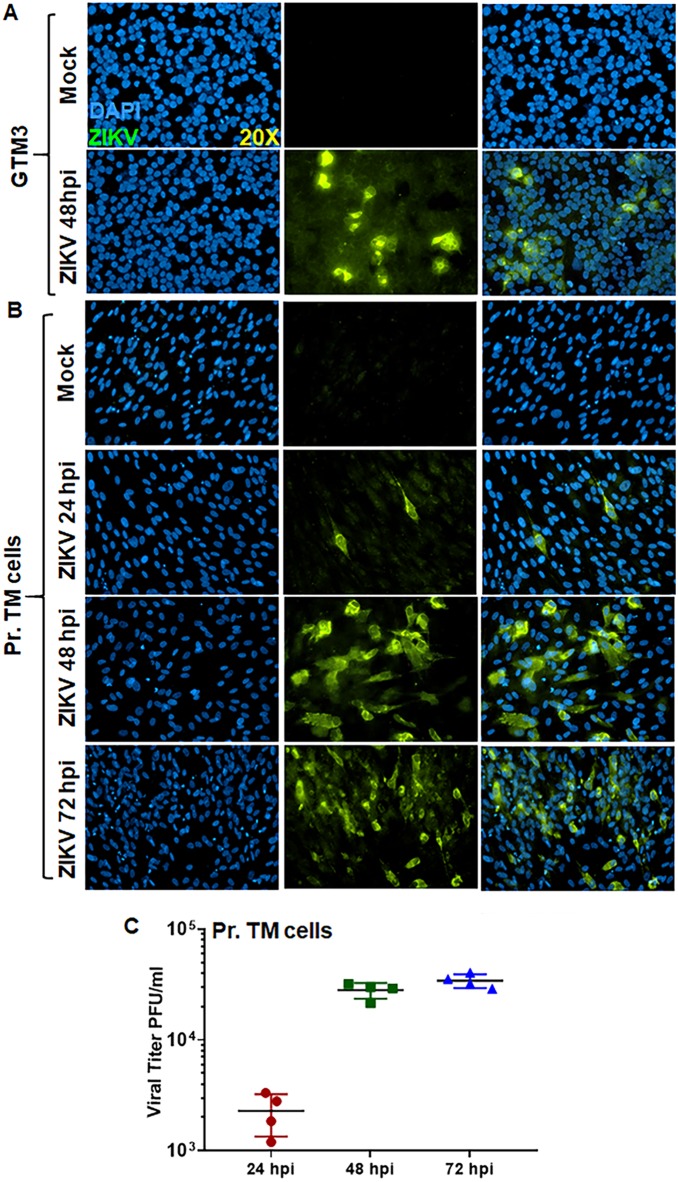
ZIKV permissively infects trabecular meshwork (TM) cells. An immortalized human TM cell line, GTM3 (A), and human primary (Pr.) TM cells (B) were infected with ZIKV (PRVABC59, a Puerto Rico strain) at MOI of 1 for indicated time points. Mock-treated cells were used as control. Infected and mock-treated cells were subjected to immunostaining for anti-flavivirus group antigen 4G2, and representative images (*n* = 3) show the presence of ZIKV (green) and DAPI (blue; a cell nuclear stain). (C) The culture supernatant of ZIKV-infected Pr. TM cells was collected and used for plaque assay on Vero cells. The dot plot represents the PFU/ml of conditioned medium (*n* = 4) at various time points.

In the eye, the TM cells maintain normal intraocular pressure (IOP) via regulating the outflow of aqueous humor, and TM dysfunction results in elevated IOP due to outflow resistance (42–44). Therefore, we determined the susceptibility of TM cells to ZIKV using human primary (Pr.) TM cells and an immortalized human GTM3 cell line. We found that ZIKV infected an immortalized human GTM3 cell line, as evidenced by a significant number of ZIKV envelope antigen (4G2)-positive cells at 48 h postinfection (Fig. 1A). A time course study in Pr. TM cells also revealed the permissive nature of TM cells, showing a temporal increase in ZIKV (4G2) positivity (Fig. 1B). Similarly to 4G2 immunostaining, the plaque assay using culture supernatants of ZIKV-infected Pr. TM cells further confirmed the replication of ZIKV in TM cells and the production of infectious progeny (Fig. 1C). These results indicated that ZIKV can infect cultured TM cells.

### ZIKV induces an innate inflammatory and antiviral response in primary TM cells.

**FIG 2:**
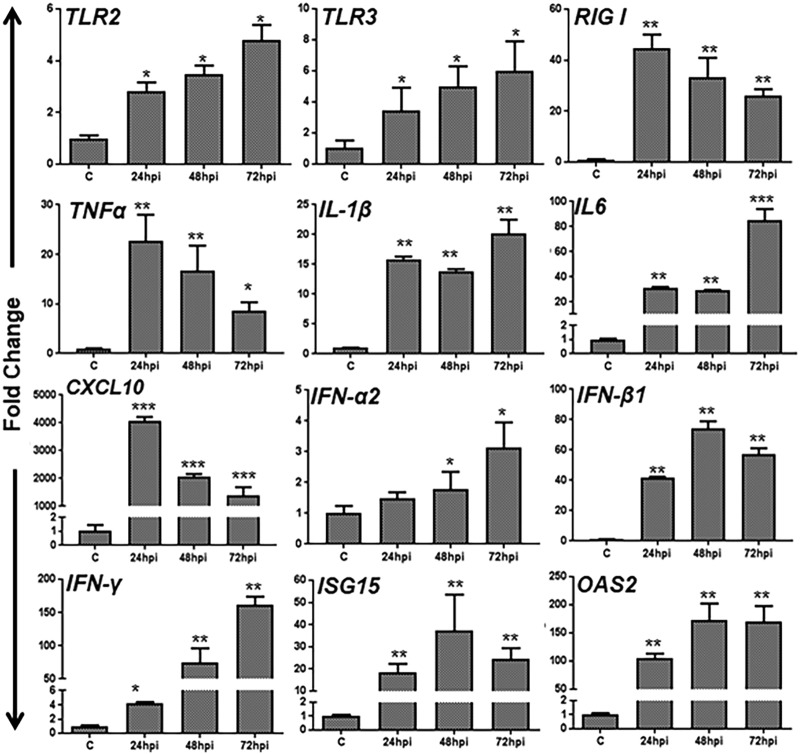
ZIKV elicited an innate antiviral immune response in primary human TM cells. Human Pr. TM cells were infected with ZIKV at MOI of 1 for indicated time points (24, 48, and 72 h postinfection [hpi]). Mock-treated cells were used as control (C). Infected and mock-treated cells were subjected to qRT-PCR for gene expression of pattern recognition receptors (PRRs) (*TLR2*, *TLR3*, and *RIG-I*), inflammatory cytokines/chemokines (*TNF-α*, *IL-1β*, *IL-6*, and *CXCL10*), type I and II interferons (*IFN-α2*, *IFN-β1*, and *IFN-γ*), and interferon-stimulated genes (*ISG15* and *OAS2*). Data represent mean ± SD. *, *P* < 0.05; **, *P* < 0.005; ***, *P* < 0.0005 (Student’s *t* test).

In order to determine whether ZIKV induces innate inflammatory and antiviral responses in TM cells, the expression of various inflammatory and antiviral genes was determined by qRT-PCR. The comparative analysis of ZIKV-infected TM cells with mock-infected cells showed the induced gene expression of classical pattern recognition receptors (PRRs), *TLR2*, *TLR3,* and *RIG-I*. The expression of *TLR2* and *TLR3* levels increased, while *RIG-I* expression peaked at 24 h postinfection (hpi) followed by a slight decrease at later time points (Fig. 2). Similarly to PRRs, ZIKV induced the gene expression of several inflammatory cytokines (*TNF-α*, *IL-1β*, and *IL-6*), chemokines (*CCL5* and *CXCL10*), and type I and II interferons (IFNs), including *IFN-α2*, *IFN-β1*, and *IFN-γ.* Concomitantly with induced IFN expression, ZIKV-infected TM cells also showed significant upregulation of interferon-stimulated genes (ISGs), including *ISG15* and *OAS2* (Fig. 2). Taken together, these results indicated that TM cells possess the ability to respond to ZIKV infection by inducing innate antiviral responses.

### ZIKV increases IOP and causes chorioretinal atrophy in mice.

**FIG 3:**
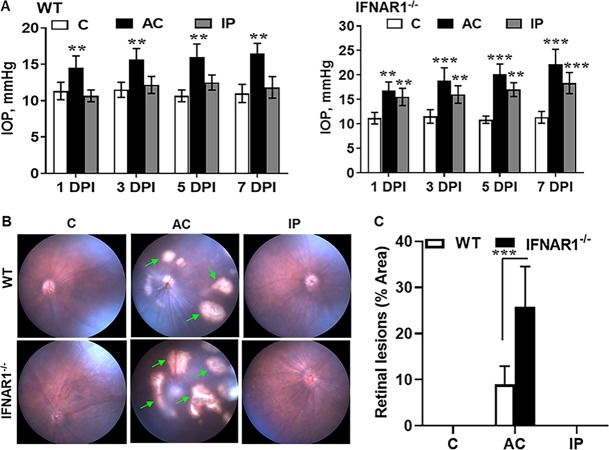
ZIKV infection elevated IOP and caused chorioretinal atrophy in mice. Eyes of 3- to 4-week-old C57BL/6 WT and IFNAR1^−/−^ mice (*n* = 6/group) were injected with ZIKV PRVABC59 either by an anterior chamber (AC) (1 × 10^4^ PFU/2 µl/eye) or by intraperitoneal (IP) (1 × 10^5^ PFU/50 µl) injection. Saline-injected mice were used as mock control (C). (A) IOP was recorded every alternate day postinfection (dpi) using a rodent TonoLab rebound tonometer. (B) Representative funduscopic images showing the retinal lesions/chorioretinal atrophy (indicated with green arrows) in WT and IFNAR1^−/−^ mice. (C) Bar graph showing quantification of ZIKV-induced retinal lesions using Image J software. Data represent mean ± SD. **, *P* < 0.005; ***, *P* < 0.0005 (Student’s *t* test).

Because ZIKV infection has been shown to increase IOP in infants (20, 21) and elevated IOP is an important risk factor for glaucoma (44, 45), we investigated the effect of ZIKV infection in IOP levels. Both WT and IFNAR1^−/−^ mice were challenged with ZIKV either by anterior chamber (AC) inoculation or by intraperitoneal (i.p.) routes. We observed that ZIKV challenge by either AC or i.p. routes resulted in a time-dependent increase in IOP in IFNAR1^−/−^ mice. In contrast, WT mice exhibited a significant increase in IOP by AC injections only (Fig. 3A). The fundus imaging of infected mice revealed that AC inoculation of ZIKV caused chorioretinal atrophy in both WT and IFNAR1^−/−^ mice (Fig. 3B), indicating ZIKV transmission from AC toward the posterior segment of the eye. IFNAR1^−/−^ mice exhibited significantly more retinal lesions than WT mice (Fig. 3B and C). In contrast, i.p. injections did not show retinal pathology in either WT or IFNAR1^−/−^ mice.

### ZIKV localizes in iridocorneal angle and TM in mice and induces TM cell death.

**FIG 4:**
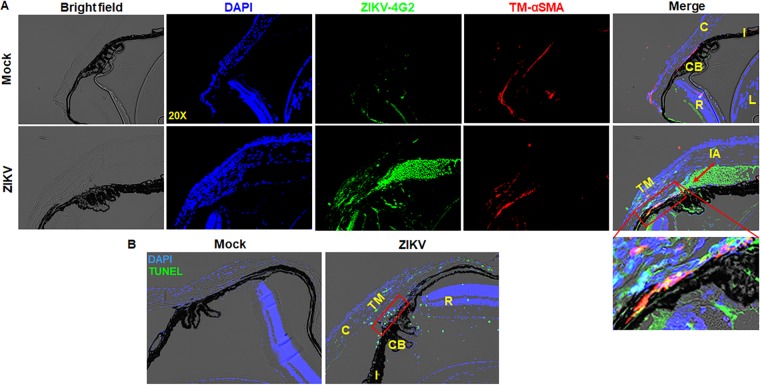
ZIKV localizes in iridocorneal angle and TM cells in mouse eyes. Eyes of 3- to 4-week-old IFNAR1^−/−^ mice (*n* = 6) were infected with ZIKV by anterior chamber (AC) (1 × 10^4^ PFU/2 µl/eye) injection. Saline-injected mice were used as mock control. (A) Iridocorneal sections of mock- and ZIKV-infected eyes were immunostained for anti-flavivirus group antigen 4G2 (ZIKV, green) and anti-smooth muscle actin (αSMA) (TM, red) and counterstained with DAPI (nuclear stain, blue). (B) Mock- and ZIKV-infected IFNAR1^−/−^ mouse iridocorneal sections were subjected to TUNEL staining. Representative images showing TUNEL-positive cells (green) and DAPI (blue). TM is shown in a red rectangle. IA, iridocorneal angle, shown with the red arrow; C, cornea; R, retina; CB, ciliary body; I, iris; L, lens.

ZIKV has been shown to be present in ciliary body and iris following systemic infection (11). Since Pr. TM cells are susceptible to ZIKV infection *in vitro*, we hypothesized that ZIKV reaches the TM cells either directly from the iris or ciliary body and/or by aqueous humor flow through the trabecular pathways and infects the TM cells. In order to investigate the interaction of ZIKV with TM *in vivo*, we challenged IFNAR1^−/−^ mice with ZIKV by the AC route. The histological analysis of ZIKV-infected eyes showed the colocalization of ZIKV envelope antigen (4G2) with α-smooth muscle actin (α-SMA), indicating the presence of ZIKV in iridocorneal angle as well as in TM (Fig. 4A). In contrast, mock-treated mice did not show viral antigen (4G2) positivity.

Viral infection often leads to cell death. Therefore, we investigated the effect of ZIKV infectivity on TM cell death *in vivo* using the terminal deoxynucleotidyl transferase dUTP nick end labeling (TUNEL) assay. Our data showed that ZIKV infection induced TM cell death, as revealed by the presence of numerous TUNEL-positive cells in TM and iridocorneal angle regions (Fig. 4B).

### ZIKV elicits an innate immune response in the anterior segment tissue of the mice.

**FIG 5:**
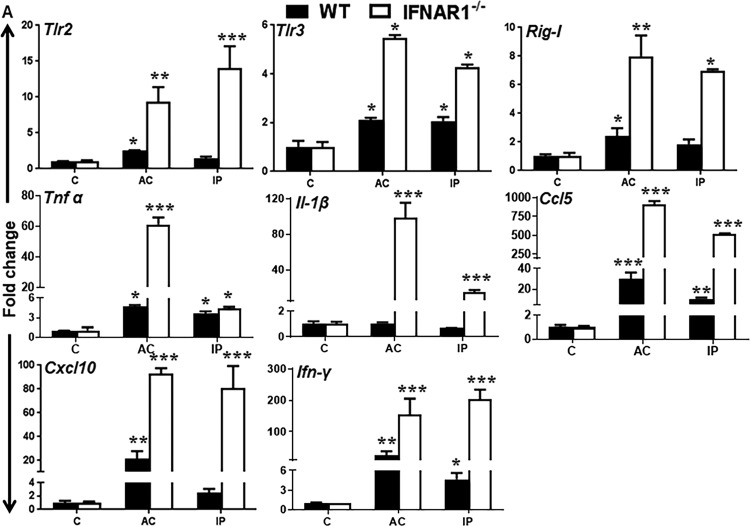
ZIKV infection induces innate antiviral immune responses in anterior segment tissue of the eye. Eyes of 3- to 4-week-old C57BL/6 WT and IFNAR1^−/−^ mice (*n* = 6/group) were injected with ZIKV either by an anterior chamber (AC) (1 × 10^4^ PFU/2 µl/eye) or by intraperitoneal (IP) (1 × 10^5^ PFU/50 µl) injections. Saline-injected mice were used as mock-treated control (C). Anterior segment tissue was harvested for RNA isolation and qRT-PCR analysis. qRT-PCR showing gene expression of PRRs (*Tlr2*, *Tlr3*, and *Rig-I*), inflammatory cytokines/chemokines (*Tnf-α*, *Il-1β*, *Ccl5*, and *Cxcl10*), and interferon (*Ifn-γ*) following ZIKV infection. Data represent mean ± SD. *, *P* < 0.05; **, *P* < 0.005; ***, *P* < 0.0005, Student’s *t* test.

Since we observed TM infectivity and cell death *in vivo* as well as induction of innate antiviral response by TM cells *in vitro*, we sought to determine whether ZIKV evoked an innate immune response in the anterior chamber of the eye. We infected WT and IFNAR1^−/−^ mice by either AC or i.p. routes and quantified the mRNA expression of genes regulating innate inflammatory and antiviral response in the anterior segment tissue of the eye at 7 days postinfection (dpi). Our results showed that mice challenged with ZIKV by either AC or i.p. injections induced the gene expression of various PRRs (*Tlr2*, *Tlr3*, and *Rig-I*) and cytokines/chemokines (*Tnf-α*, *Il-1β*, *Ccl5*, *Cxcl10*, and *Ifn-γ*) (Fig. 5). Moreover, expression levels of these transcripts were higher in IFNAR1^−/−^ than in WT anterior segment tissue.

### ZIKV infects RGCs and induces RGC death and loss in mice.

**FIG 6:**
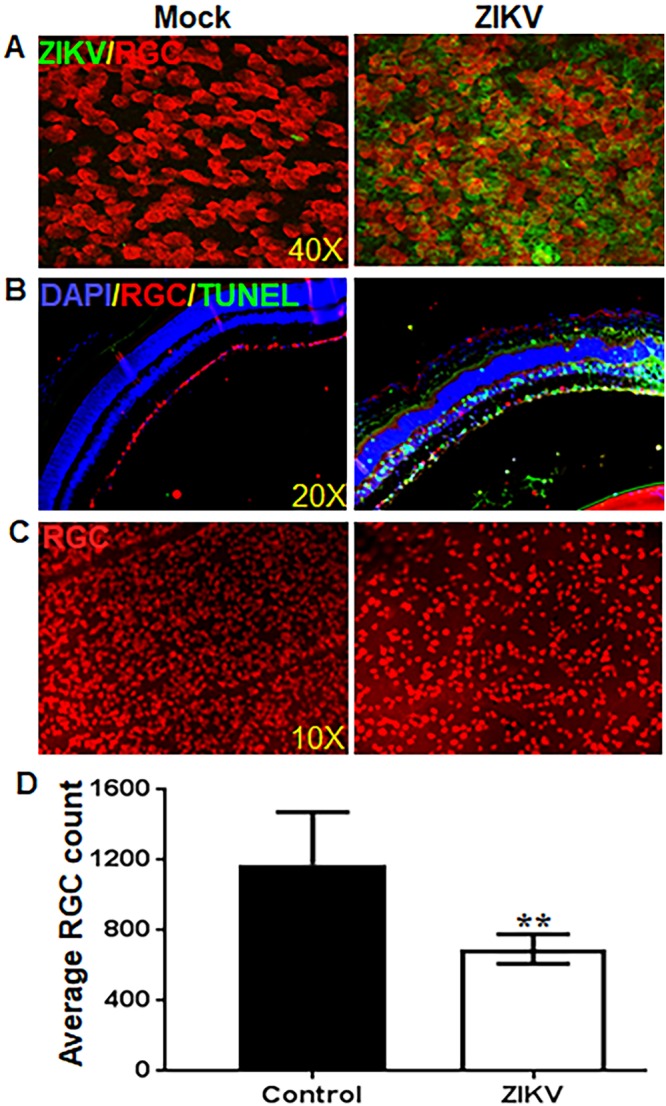
ZIKV infects retinal ganglion cells (RGCs) and causes cell death. Eyes of 3- to 4-week-old IFNAR1^−/−^ mice (*n* = 6) were infected with ZIKV by anterior chamber (AC) (1 × 10^4^ PFU/2 µl/eye) injection. Saline-injected mice were used as mock control. (A) Mock- and ZIKV-infected mouse whole-mount retinas were subjected to RBPMS (RGC, red) and anti-flavivirus group antigen 4G2 (ZIKV, green) staining. (B) Mock- and ZIKV-infected mouse eye cryosections were subjected to TUNEL staining followed by immunostaining for RBPMS. Representative images (*n* = 6) showing TUNEL-positive cells (green) and RGCs (red). (C) Mock- and ZIKV-infected IFNAR1^−/−^ mouse retinas were subjected to whole-mount staining for RGCs (RBPMS, red). (D) The bar graph represents the average RGC count per retina (a total of 16 frames covering the density range of 300 to 1,500 RGCs per frame were counted using automated software). Data represent mean ± SD (**, *P* < 0.005; *t* test, *n* = 6).

Because we observed the development of retinal lesions in eyes infected with ZIKV (Fig. 3) and the death and loss of RGCs are considered a hallmark of glaucoma (45), we investigated the effect of ZIKV infection on RGC health. IFNAR1^−/−^ mice were infected with ZIKV via AC route, and retinal whole-mount immunostaining was performed to visualize RGCs using a specific cell marker, RNA-binding protein with multiple splicing (RBPMS). Our data showed that ZIKV infected RGCs in the retina as revealed by colocalization of 4G2 and RBPMS in retinal flat mounts (Fig. 6A). We also observed numerous TUNEL-positive cells localized with RBPMS all across the ganglion cell layer (GCL), indicating the death of RGCs in ZIKV-infected mouse eyes (Fig. 6B). In addition to GCL, inner nuclear layer (INL) also showed the presence of TUNEL-positive cells. In contrast, 4G2 positivity was not observed in mock-treated mice and the uniform RBPMS staining throughout the GCL indicated intact/healthy RGCs in these eyes. Furthermore, the counting of RGCs (RBPMS fluorescence-positive cells) in retinal whole mounts revealed a significant loss of RGCs in ZIKV-infected mouse retina (Fig. 6C and D).

### ZIKV causes disruption of anterograde axonal transport in the ON.

**FIG 7:**
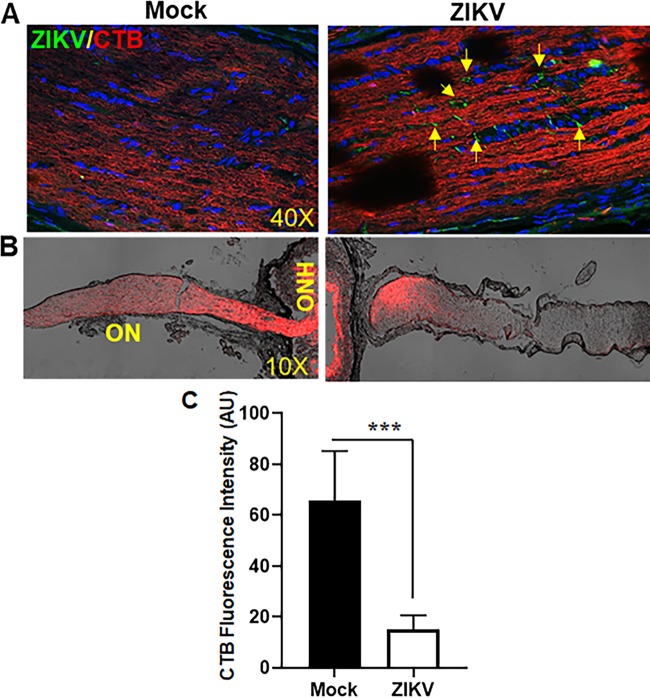
ZIKV infects optic nerve and disrupts anterograde axonal transport. Eyes of 3- to 4-week-old IFNAR1^−/−^ mice (*n* = 6) were infected with ZIKV by anterior chamber (AC) (1 × 10^4^ PFU/2 µl/eye) injection. Saline-injected mice were used as mock control. Five days after ZIKV infection, mice were injected intravitreally with Alexa Fluor 594-labeled cholera toxin B (CTB) (2 µg/2 µl/eye), and 48 h following CTB injection, optic nerve was surgically detached from the superior colliculus from the brain and cryosectioned. (A) Optic nerve cryosections were subjected to immunostaining for anti-flavivirus group antigen 4G2 (ZIKV, green), and representative confocal images show ZIKV optic nerve head infection (yellow arrows). (B) Optic nerve cryosections were imaged for CTB fluorescence (red) intensity across the optic nerve as an indicator of axonal transport. (C) The bar graph represents quantification of CTB fluorescence intensity across the optic nerve using Image J software. Data represent mean ± SD (***, *P* < 0.0005; *t* test; *n* = 6). ON, optic nerve; ONH, optic nerve head.

Vision loss in glaucoma ascends from the degeneration of RGCs neurons and their axons, comprising the optic nerve (ON) (45, 46). Studies indicate that a functional deficit in anterograde axon transport along the ON precedes degeneration of RGC axons in glaucoma (46–48). We observed that AC inoculation with ZIKV led to the development of chorioretinal atrophy and retinal cell death, including RGCs and their loss. Therefore, we hypothesized that ZIKV is transmitted vertically from AC toward the posterior segment of the eye, causing damage to the retina and the optic nerve. To assess the impairment of ON function, we measured anterograde transport by intravitreal injection of cholera toxin subunit B (CTB), which labels entire retinal projection via active uptake and transport (46, 47). As shown in Fig. 7A, AC inoculation with ZIKV in IFNAR1^−/−^ mice led to ON infectivity as evident by the immunofluorescence staining of viral envelope antigen 4G2. Moreover, ZIKV disrupted the anterograde transport in ON as evidenced by reduced fluorescence of CTB across the longitudinal section of the ON, an indication of damaged ON axons (Fig. 7B and C) in ZIKV-infected eyes. In contrast, intact CTB fluorescence is evident in the ON in mock-treated mice, indicating normal axonal transport.

## DISCUSSION

Glaucoma is primarily considered a genetic and age-related eye disease. However, certain inflammatory conditions, such as uveitis and virus-induced anterior segment inflammation, can predispose to glaucoma (49, 50). Indeed, several viruses, such as herpes simplex virus (HSV), varicella-zoster virus (VZV), Epstein-Barr virus (EBV), and cytomegalovirus (CMV), have been linked to causing either primary or secondary glaucoma (50–53). Recently, several clinical case reports from ZIKV-affected countries have linked ZIKV infection to causing congenital glaucoma. Apart from these clinical correlations, to the best of our knowledge, the role of ZIKV in causing glaucoma pathobiology is not known. While the underlying molecular mechanisms are still being investigated, our study provides new insights into the pathogenesis of ZIKV in causing glaucoma.

TM cells are the key cells involved in the regulation of aqueous humor outflow and maintaining normal IOP. While TM cells have been shown to be susceptible to several viruses (50, 54, 55), the ability of the viruses to invade and permissively infect TM cells remains poorly understood. Here, we reported that Pr. TM cells and an immortalized human TM cell line, GTM3, were permissive to ZIKV infection. Some studies suggest that TM cells have the ability to produce inflammatory mediators in response to growth factors and cytokine challenge (56, 57), mechanical stress (43), and CMV infections (42). Similarly, we found that ZIKV-infected TM cells elicited a potent innate immune response characterized by induced expression of various PRRs, multiple cytokines/chemokines, IFNs, and ISGs. These findings indicate the ability of TM cells to respond to ZIKV infection.

**FIG 8:**
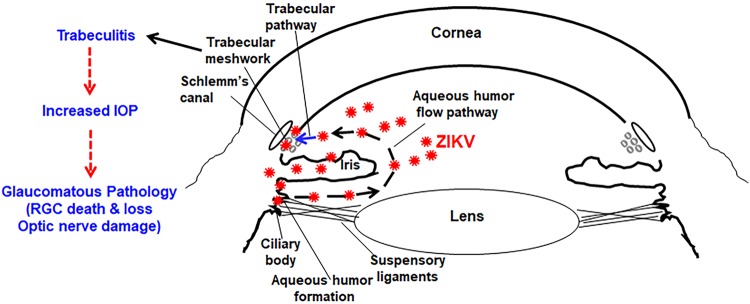
Schematic model of ZIKV-induced glaucoma. ZIKV has been shown to be present in ciliary body and iris from systemic circulation following infection. We hypothesize that ZIKV challenge, either by anterior chamber inoculation or by intraperitoneal/subcutaneous routes, reaches the TM cells either directly from the iris or ciliary body or by aqueous humor flow through trabecular pathways, infects TM cells, and induces trabeculitis, leading to increased IOP and glaucomatous pathology, *viz*., RGC death and loss and optic nerve damage.

Clinical studies of congenital ZIKV infection have shown elevated IOP in infants (20), which is also an important risk factor for glaucoma in adults. In our study, we observed that ZIKV infection caused elevated IOP in both IFNAR1^−/−^ and WT mice. Moreover, AC inoculation with ZIKV resulted in the development of chorioretinal atrophy, indicating viral transmission from the anterior segment toward the posterior/retina of the eye. Our data also showed that ZIKV infection by a systemic route (i.p.) resulted in elevated IOP in IFNAR1^−/−^ but not the WT mice. Indeed, an increase in IOP has been reported with ZIKV infection in IFN-α/βR^−/−^ and WT mice (58). Similarly, viral infections in humans, including those caused by HSV, herpes zoster virus (HZV), EBV, and CMV, have been shown to increase IOP (59–62). Whether the increased IOP observed in our study is due to general viral infection or is specific to ZIKV needs further investigation by challenging the mice with viruses closely related to ZIKV, e.g., dengue virus (25, 63), or unrelated viruses such as VZV. However, based on our *in vitro* and *in vivo* findings of susceptibility of TM cells to ZIKV and the induction of an inflammatory response, we propose that ZIKV-induced trabeculitis could be one of the potential mechanisms for increased IOP and glaucomatous pathology (Fig. 8).

Another pathophysiological hallmark of glaucoma is RGC death and loss. Several studies have shown RGCs or their axonal loss in experimental models of glaucoma (64–66). We found that ZIKV infection caused RGC death, resulting in their significant loss in IFNAR1^−/−^ mice. Because the optic nerve consists of RGC axons, the loss of RGCs in glaucoma impairs axonal transport (46, 48, 67, 68). Although ZIKV has been shown to infect the optic nerve head in neonate pups (6), our study is the first to demonstrate the disruption of anterograde axonal transport in ZIKV-infected eyes. These findings support our hypothesis that ZIKV can be vertically transmitted from the anterior to the posterior segment of the eye toward the optic nerve and cause retinal atrophy and retinal cell death, including that of RGCs. Because we observed both an increased IOP and RGC death in our experimental model, one of the key questions is whether RGC death or loss is due to ZIKV-induced IOP or direct infection. We think that, because of an acute infection model (7 dpi), the RGC death is unlikely to be due to increased IOP but rather is due to direct killing of RGCs by ZIKV. However, it would be interesting to investigate this for a longer duration of ZIKV infection, when there will be no circulating virus. Therefore, further studies are needed to elucidate mechanisms of RGC death during ZIKV infection.

In summary, our study showed that human primary TM cells support ZIKV replication and elicit an innate immune response by activating PRRs and producing inflammatory cytokines, interferons, and ISGs. Moreover, in mouse eyes, ZIKV infection resulted in TM infection and cell death, increased IOP, and chorioretinal atrophy. Furthermore, ZIKV-infected mice exhibited glaucomatous pathology characterized by RGC death and loss and disruption of axonal transport. Given the limited studies linking infectious causes of glaucoma, our study may provide a basis for future research in this area.

## MATERIALS AND METHODS

### Cells and culture conditions.

Human primary trabecular meshwork (TM) (69, 70) cells and the human GTM3 cell line (69) were cultured in Dulbecco’s modified Eagle’s medium (DMEM) supplemented with 10% fetal bovine serum (FBS), 10 µg/ml l-glutamine, and 1% penicillin-streptomycin (Thermo Scientific, Rockford, IL) solution. Vero cells (ATCC CCL-81) and Aedes albopictus clone C6/36 cells (ATCC CRL-1660) were cultured in DMEM and Eagle’s minimal essential medium (EMEM), respectively, supplemented with 10% FBS, 10 µg/ml l-glutamine, and 1% penicillin-streptomycin solution per the manufacturer’s recommendation. All the cells (except C6/36, maintained at 28°C) were maintained at 37°C with 5% CO_2_. The plaque assay was performed using Vero cells as described in one of our recent studies (25).

### Mice and ethics statement.

C57BL/6 (wild type [WT]) and type I interferon receptor α chain-knockout (IFNAR1^−/−^) mice were purchased from Jackson Laboratory (Bar Harbor, ME) and bred in-house. Animals were housed in a restricted-access Division of Laboratory Animal Resources (DLAR) facility, maintained on a 12-h light–12-h dark cycle, and fed on LabDiet rodent chow (PicoLab; LabDiet, St. Louis, MO) and water *ad libitum*. Both male and female mice, 3 to 4 weeks of age, were used in all experiments. Mice were treated in compliance with the Association for Research in Vision and Ophthalmology (ARVO) Statement for the Use of Animals in Ophthalmic and Vision Research, and all procedures were approved by the Institutional Animal Care and Use Committee (IACUC) of Wayne State University.

### Virus strains and infection.

Zika virus (ZIKV) strain PRVABC59, NR-50240 (12, 63), originally isolated from human blood in Puerto Rico in December 2015, was obtained through BEI Resources, NIAID, NIH. ZIKV was propagated in C6/36 and ATCC CCL-81 Vero cell lines, and titers were determined by standard plaque assays as described previously (25). All cells in *in vitro* experiments were infected with ZIKV at MOI of 1 unless specified. For *in vivo* studies, both WT and IFNAR1^−/−^ mice were injected with ZIKV either by an anterior chamber (AC) (1 × 10^4^ PFU/2 µl/eye) or by intraperitoneal (i.p.) (1 × 10^5^ PFU/50 µl) injections.

### IOP measurements and fundus imaging.

An Icare TonoLab rebound tonometer (Colonial Medical Supply, Windham, NH), a rodent-dedicated tonometer, was used for measuring intraocular pressure (IOP). IOP was determined in behaviorally trained conscious mice as described by Clark and coworkers (71). All IOP measurements were performed between 10 a.m. and 12 p.m. An average of 6 individual IOP measurements were taken to calculate the average final IOP value for each eye. All IOP measurements were recorded in a masked manner.

Fundus images were recorded using a Micron III camera per the manufacturer’s recommendations (Phoenix Research Labs, Pleasanton, CA).

### Anterograde transport measurements.

Mice were anesthetized using a ketamine (67 mg/kg of body weight)-xylazine (10 mg/kg) mixture and injected intravitreally with Alexa Fluor 594-conjugated cholera toxin subunit B (CTB) (2 µg/2 µl/eye) using a 34G needle. Two days following CTB injections, mice were transcardially perfused with PBS followed by 4% paraformaldehyde (PFA) in PBS. Following perfusion, the optic nerve was detached from superior colliculus from the brain and fixed in 4% PFA overnight, followed by incubation with sucrose gradient (10 to 30% [wt/vol]) and embedded in OCT. Five- to 6-µm thin optic nerve cryosections were collected and imaged for the transport of CTB, by visualizing CTB fluorescence intensity across the optic nerve using a confocal microscope. Optic nerve cryosections were also immunostained for ZIKV envelope antigen as described below.

### Immunostaining.

Immunostaining procedures were performed as described previously (12, 25, 63). Briefly, human Pr. TM and GTM3 cells were cultured in a four-well chamber slide (Fisher Scientific, Rochester, NY) and infected with ZIKV at MOI of 1. At desired time points, ZIKV-infected and mock-treated cells were fixed overnight with 4% PFA in PBS at 4°C. After washing with 1× PBS, the cells were permeabilized and blocked with 1% (wt/vol) BSA and 0.4% Triton X-100 made in PBS for 1 h at room temperature (RT). Cells were incubated with primary mouse monoclonal antibody 4G2 (1:100) (Millipore, Billerica, MA; catalog number MAB10216), overnight at 4°C. Following removal of the primary antibody, the cells were washed extensively with PBS and incubated for 1 h with anti-mouse/rabbit Alexa Fluor 485/594-conjugated secondary antibody (1:200) at RT. Finally, the cells were extensively washed with PBS and the slides were mounted in Vectashield antifade mounting medium (Vector Laboratories, Burlingame, CA) and visualized using an Eclipse 90i fluorescence microscope (Nikon, Melville, NY).

For *in vivo* experiments, mouse eyes were enucleated and fixed in 4% PFA, dehydrated, and embedded in paraffin. Ten-micrometer thin sections were made using a cryostat and mounted onto lysine-coated microscope slides. For immunostaining, iridocorneal sections, as well as retinal sections, were fixed in 4% PFA for 20 min at RT followed by four washes with PBS (10 min for each wash). Eye sections were permeabilized, blocked with 10% normal goat serum with 0.5% Triton X-100 for 2 h at RT, and incubated overnight with primary antibody (1:100). Next day, sections were washed four times with PBS (10 min each) and incubated with anti-mouse/rabbit Alexa Fluor 485/594-conjugated secondary antibody (1:200) for 2 h at RT. The eye sections were extensively washed with PBS (4 washes, 10 min each), and the slides were mounted in Vectashield antifade mounting medium (Vector Laboratories, Burlingame, CA) and visualized using either an Eclipse 90i fluorescence microscope (Nikon, Melville, NY) or a Keyence microscope (Keyence, Itasca, IL).

### Retinal whole-mount staining.

Mice were euthanized, and eyes were enucleated with a pair of forceps. The eyeballs were fixed in 4% PFA for 24 h at 4°C with gentle shaking. Following fixation, cornea/iris tissue was removed surgically and the retina was isolated carefully under a dissection microscope. The retina was washed three times with PBS in a 48-well plate and permeabilized and blocked simultaneously using 10% normal goat serum with 0.5% Triton X-100 overnight at 4°C. The retinal tissue was incubated overnight with primary RBPMS/4G2 antibody (1:100) diluted in 10% goat serum with 0.5% Triton X-100. Next day, retinal tissue was washed four times (10 min for each wash) and incubated overnight with anti-rabbit/mouse–Alexa Fluor 594/485 antibody (1:200). On the following day, retinal tissues were washed four times (10 min for each wash), flattened with four partial cuts from the limbal to central retina, mounted onto a glass slide using Vectashield mounting medium (Vector Laboratories, Burlingame, CA), and visualized under a Leica SP8 confocal microscope (Leica Microsystems, Buffalo Grove, IL).

### TUNEL assay.

Mouse eyes were enucleated and fixed in 4% PFA for 24 h at 4°C. Following fixation, eyes were passed through a gradient of sucrose (10% to 30% [wt/vol]) and embedded in Tissue-Tek OCT (Sakura, Torrance, CA). Ten-micrometer thin sections were made using a cryostat and mounted onto lysine-coated microscope slides. Terminal deoxynucleotidyl transferase dUTP nick end labeling (TUNEL) staining was performed using the ApopTag fluorescein *in situ* apoptosis detection kit per the manufacturer’s instructions (Millipore, Billerica, MA). The TUNEL-stained cells were visualized using either an Eclipse 90i fluorescence microscope (Nikon, Melville, NY) or a Keyence microscope (Keyence, Itasca, IL).

### RNA extraction and qRT-PCR.

Total RNA was extracted from ZIKV-infected TM cells and anterior segment tissue from infected and control mice using TRIzol per the manufacturer’s recommendation (Thermo Scientific, Rockford, IL). cDNA was synthesized using 1 µg of total RNA using a Maxima first-strand cDNA synthesis kit, per the manufacturer’s instructions (Thermo Scientific, Rockford, IL). The cDNA was amplified using gene-specific PCR primers. qPCR was conducted in a StepOnePlus real-time PCR system (Applied Biosystems, Grand Island, NY) using TaqMan probes against genes expressing various inflammatory cytokines/chemokines (*TNF-α*, *IL-1β*, *IL-6*, *CXCL10*, and *CCL5*), type I and II interferons (*IFN-α2*, *IFN-β1*, and *IFN-γ*), and Toll-like receptors (*TLRs*) and SYBR green-based primers against *RIG-I* and IFN-stimulated genes like *ISG15, OAS2*, and *MX1*. All primers and TaqMan probes (Prime Time mini-qPCR assay) were purchased from Integrated DNA Technologies (Coralville, IA, USA). The quantification of gene expression was determined via the comparative threshold cycle (ΔΔ*C_t_*) method. Gene expression in the test samples was normalized to the endogenous reference GAPDH level and was reported as fold change relative to GAPDH gene expression.

### Statistics.

The data have been expressed as the mean ± standard deviation (SD) unless indicated otherwise. Statistical differences between experimental groups were determined using unpaired Student’s *t* test. All statistical analyses were performed using GraphPad Prism 7 (GraphPad Software, La Jolla, CA). A *P* value of <0.05 was considered statistically significant. All experiments were performed at least three times unless indicated otherwise.
